# Eukaryotic Elongation Factor 3 Protects *Saccharomyces cerevisiae* Yeast from Oxidative Stress

**DOI:** 10.3390/genes11121432

**Published:** 2020-11-28

**Authors:** Karolina Gościńska, Somayeh Shahmoradi Ghahe, Sara Domogała, Ulrike Topf

**Affiliations:** Institute of Biochemistry and Biophysics, Polish Academy of Sciences, 02-106 Warsaw, Poland; kgoscinska@ibb.waw.pl (K.G.); s.shahmoradi@ibb.waw.pl (S.S.G.); sara.domagala123@gmail.com (S.D.)

**Keywords:** eEF3, *HEF3*, *YEF3*, oxidative stress, reactive oxygen species (ROS), translation, elongation, fungi

## Abstract

Translation is a core process of cellular protein homeostasis and, thus, needs to be tightly regulated. The production of newly synthesized proteins adapts to the current needs of the cell, including the response to conditions of oxidative stress. Overall protein synthesis decreases upon oxidative stress. However, the selective production of proteins is initiated to help neutralize stress conditions. In contrast to higher eukaryotes, fungi require three translation elongation factors, eEF1, eEF2, and eEF3, for protein synthesis. eEF1 and eEF2 are evolutionarily conserved, but they alone are insufficient for the translation elongation process. eEF3 is encoded by two paralogous genes, *YEF3* and *HEF3*. However, only *YEF3* is essential in yeast, whereas the function of *HEF3* remains unknown. To elucidate the cellular function of Hef3p, we used cells that were depleted of *HEF3* and treated with H_2_O_2_ and analyzed the growth of yeast, global protein production, and protein levels. We found that *HEF3* is necessary to withstand oxidative stress conditions, suggesting that Hef3p is involved in the selective production of proteins that are necessary for defense against reactive oxygen species.

## 1. Introduction

Protein synthesis (translation) is an essential cellular process that is highly regulated to preserve homeostasis and react to current protein demands of the cell. Translation relies on many intrinsic and extrinsic factors, including energy availability and environmental stress [1]. Mechanistically, the ribosome serves as a macromolecular machine that decodes information that is encoded in mRNA and synthesizes polypeptide chains. Although the ribosome is an intricate ribonucleoprotein complex, it needs assistance from other proteins, known as translation factors, in each phase of the protein synthesis process. Translation factors are multifunctional, soluble proteins. Their involvement increases the fidelity, speed, and accuracy of protein production [2]. Moreover, they play other roles beyond the protein synthesis process, such as in virus replication and cancer development [3,4].

Distinct translation factors cooperate with the ribosome at each stage of protein synthesis. The mechanism and involvement of translation factors also differ between prokaryotes and eukaryotes, especially at the level of translation initiation, termination, and ribosome recycling [5]. The initiation of protein synthesis is a common rate-limiting step and is thus tightly regulated [6]. In eukaryotes, at least 12 translation factors are required to initiate mRNA translation [7,8,9]. In contrast, during subsequent stages of protein synthesis, the ribosome requires less support from translation factors [5]. Only two release factors take part in the termination of protein synthesis, eRF1 (YBR143C), which recognizes stop codons, and eRF3 (YDR172W) exhibiting GTPase activity [10]. Ribosome recycling machinery utilizes three translation factors which are involved in the translation initiation stage: eIF1 (YNL244C), eIF1A (YMR260C), and eIF3 complex with loosely associated eIF3j (YLR192C) subunit [11]. The most conserved step of protein synthesis is the elongation of the polypeptide chain. It consists of three stages that are mediated by two elongation factors in higher eukaryotes, eEF1 (YBR118W/YPR080W) and eEF2 (YJR047C/YEL034W). In the first stage, one subunit of the eEF1 complex, eEF1A associates with GTP and delivers aminoacyl-tRNA to the decoding site (i.e., the A-site) on the ribosome [12]. Following proper codon-anticodon binding, eEF1A dissociates in a complex with guanosine diphosphate (GDP) and is recovered by eEF1B (YAL003W) exchange factor to the GTP-bound form to bind another tRNA [13]. The second stage of translation elongation involves the formation of the peptide bond that occurs in the “hybrid” state of the ribosome, during which a tRNA shifts the A-site and peptidyl site (P-site) together [14]. Furthermore, the consecutive elongation factor eEF2 binds to the A-site and translocates tRNA to canonical P- and exit (E)-sites. In the third stage of translation, the release of deacylated tRNA from the E-site opens the A-site for another round of the elongation cycle.

Importantly, an additional and unique factor, eukaryotic elongation factor 3 (eEF3; YLR249W), is required by fungi for translation elongation. Mammalian eEF1 and eEF2 alone are unable to promote protein synthesis in yeast [15]. Recently, eEF3-like homologues were shown to be more widely distributed among unicellular eukaryotes (e.g., *Phytophthora infestans* oomycetes) [16,17]. eEF3 has multiple functions in the process of polypeptide chain elongation, including stimulation and checking the correctness of aminoacyl-tRNA delivery by eEF1A, branching 40S and 60S ribosome subunits, and facilitating deacyl-tRNA removal from the E-site through adenosine triphosphatase (ATPase) activity [18,19,20,21]. Some studies also suggest a role for eEF3 in ribosome recycling, particularly in disassembling the translation post-termination complex that consists of mRNA, tRNA, and the ribosome [19,22,23]. Moreover, eEF3 interacts with non-coding regions of mRNA independently of its involvement in the translation process [24].

eEF3 is encoded by two paralogous genes, *YEF3* (YLR249W) and *HEF3* (YNL014W). Yef3 is an essential protein for the viability of baker’s yeast, *Saccharomyces cerevisiae* [25]. It is a member of the ATP-binding cassette F (ABCF) ATPase family, consisting of two ABC-type ATPase domains [17,19]. The paralog of *YEF3*, *HEF3* (homolog of elongation factor 3), arose from an ancient whole-genome duplication event [26,27]. The sequences of Yef3p and Hef3p are highly conserved, with 84% sequence identity [28]. Knowledge of Hef3p is very limited, and its biological role is unknown. Hef3p is not essential for the mating, sporulation, or vegetative growth of yeast [29]. It was shown to be expressed under zinc-deficiency conditions [30]. Studies from the late 1990s showed a lack of functional complementarity between *HEF3* and *YEF3* [28,29]. This is consistent with accumulating evidence of the functional divergence of protein paralogs [31,32,33]. Interestingly, Yef3p has approximately two-fold higher ribosome-dependent ATPase activity than Hef3p, but they exhibit similar basal ATPase activity and, notably, affinity for the ribosome [28]. Considering the significance of Yef3p in fungal protein synthesis, the function of Hef3p, characterized by high similarity to Yef3p, appears to be an interesting target for further investigation.

In the present study, we investigated the role of *HEF3* during conditions of oxidative stress. We found that *HEF3* was necessary to withstand oxidative stress, and this function occurred mostly independently from its paralog, *YEF3*. Our data suggest that Hef3p is involved in regulating the expression of enzymes that are important for the detoxification of reactive oxygen species (ROS) at the level of translation rather than transcription. Thus, we identified a previously unknown function of *HEF3.* Our findings provide yet another example of paralogous genes with diverse functions that increase ribosome heterogeneity and regulate translational output.

## 2. Materials and Methods

### 2.1. Yeast Strains and Growth Conditions

BY4741 and *hef3*Δ yeast strains were obtained from Euroscarf (http://www.euroscarf.de/index.php?name=News), and the deletion strain was confirmed by amplification of the kanamycin cassette and sequencing. Yeast cells were maintained on YPD (1% yeast extract, 2% bacto peptone, and 2% glucose) plates. Yeast strains were grown in YP (1% yeast extract and 2% bacto peptone) liquid medium that contained 2% glucose or 3% glycerol as the carbon source. Generally, yeast cells were grown to an optical density at 600 nm (OD_600_) of approximately 0.6–0.8 at 28 °C in liquid culture before processing for further experiments.

### 2.2. Oxidative Stress Assays

Yeast cells were treated with different concentrations of hydrogen peroxide (H_2_O_2_; Sigma, St. Louis, MO, USA) to induce oxidative stress. Yeast cells were grown on a minimal synthetic medium (0.67% (*w*/*v*) yeast nitrogen base, 0.64% (*w*/*v*) complete amino acid mix, 2% glucose, or 2% galactose, or 2% sucrose. Two percent agar was added for growth tests on solid plates). Yeast cells in liquid cultures were grown to an OD_600_ of approximately 0.6–0.8 before adding H_2_O_2_ for 30 min. For the growth assays on solid medium, plates that contained H_2_O_2_ were freshly prepared not more than 24 h before plating yeast cells.

### 2.3. Generation of Expression Constructs

The *HEF3* gene was amplified from genomic DNA from the BY4741 wild-type yeast strain using the following primers: 5′-CAAAAAAAAAGTAAGAATTTTTGAAAATTCCAATCTAATAGAGAAGGG-3′ and 5′-CGTCATCCTTGTAATCCATCGATACTAGTGCAAAATCTTCATCAGAAGAAACG-3′. The polymerase chain reaction (PCR) product was cloned into the pESC-URA vector in frame to the FLAG-tag at the C-terminus of Hef3p using the recombination technique with the In-Fusion HD EcoDry Cloning Kit (TaKaRa, Kyoto, Japan) according to the manufacturer’s instructions. The resulting construct expresses Hef3-FLAG under the Gal1-Gal10 inducible promoter (pUT01).

### 2.4. Electrophoresis and Western Blot Analysis

Total protein extracts from 1–2.5 OD600 units of yeast cells were prepared as described previously [34] with minor modifications. Briefly, the yeast cell pellets were resuspended in cold double-distilled H_2_O (ddH_2_O), and 300 mM sodium hydroxide was added. Samples were mixed and incubated for 10 min on ice. Next, the samples were precipitated with 7% trichloroacetic acid. The samples were then vortexed, incubated for 15 min on ice, and centrifuged at 20,000× *g* for 10 min at 4 °C. The supernatants were discarded, and the pellets were washed with ice-cold acetone. The samples were centrifuged as described above, and the pellets were solubilized in Laemmli buffer that contained 50 mM DTT. Proteins were denatured at 65 °C for 15 min, and a sample volume that corresponded to 0.1 or 0.2 OD_600_ was separated on 15% sodium dodecyl sulfate-polyacrylamide gel electrophoresis (SDS-PAGE) gels. This analysis was followed by Western blot using specific antibodies. Primary antibodies were custom-raised in rabbits, and their specificity was controlled individually. Commercially available anti-FLAG M2 primary antibody (catalog no. F1804, Sigma, St. Louis, MO, USA, diluted 1:1000) and anti-rabbit IgG secondary antibody (catalog no. A9169, Sigma, St. Louis, MO, USA, diluted 1:10,000) were used. Chemiluminescence protein signals were detected of X-ray films. The images were digitally processed using free GIMP (https://www.gimp.org/) software. Densitometry measurements were performed to quantify signals from western blot analysis with ImageJ software (NIH, Bethesda, Maryland, USA). The statistical analysis was performed using one-way analysis of variance (ANOVA) followed by Tukey’s post hoc test.

### 2.5. Translation Assay

Yeast strains were grown on selective minimal synthetic medium (0.67% (*w*/*v*) yeast nitrogen base and 0.75% (*w*/*v*) Met amino acid mix) supplemented with 2% (*v*/*v*) glucose and treated with different concentrations of H_2_O_2_ (Sigma, St. Louis, MO, USA). Simultaneously with H_2_O_2_ treatment, the proteins were radiolabeled using [^35^S]-labeled methionine (catalog no. SRM-01H, Hartmann Analytic, Braunschweig, Germany) at a final concentration of 10 μCi ml^−1^ for 30 min. Yeast cells were harvested by centrifugation and washed once with ddH_2_O. Proteins were extracted by alkaline lysis for 5 min at room temperature. The obtained protein pellets were solubilized in Laemmli buffer that contained 50 mM DTT, denatured for 15 min at 65 °C, and analyzed by SDS-PAGE and digital autoradiography. Autoradiography signals were detected using Fujifilm FLA-7000 (GE Healthcare, Bio-Sciences AB, Uppsala, Sweden).

### 2.6. RNA Isolation and Quantitative PCR

Total RNA was isolated from 20 OD_600_ units of exponentially grown cells by hot phenol and the SDS method as described previously [35]. Before performing real-time PCR, 40 ng of RNA was reverse transcribed using the QuantiTect reverse transcriptase kit (Qiagen, Hilden, Germany). Oligodeoxynucleotide primers were designed by the SGD’s Primer Design tool and are shown in Table 1. Quantitative real-time PCR was performed using the Roche LightCycler 480 System (Basel, Switzerland) with RT PCR Mix SYBR C (A&A Biotechnology, Gdynia, Poland). The reaction program consisted of 5 min of initial denaturation at 95 °C, followed by 40 cycles of 30 s at 95 °C, 20 s at 55 °C, and 20 s at 72 °C, and the fluorescence intensity was read. Each sample was loaded in triplicate on a plate that contained negative controls and cDNA dilutions to generate a standard curve. After amplification, the melt curve profile of the PCR products was analyzed to ensure the lack of variation among the products. The data are presented as the relative expression of each gene to the geometric mean of three reference genes (*ACT1*, *ALG9*, and *TDH1*). The statistical analysis was performed using one-way analysis of variance (ANOVA) followed by Tukey’s post hoc test.

## 3. Results

### 3.1. Hef3p Is Not Required under Normal Vegetative Growth Conditions

**Figure 1:**
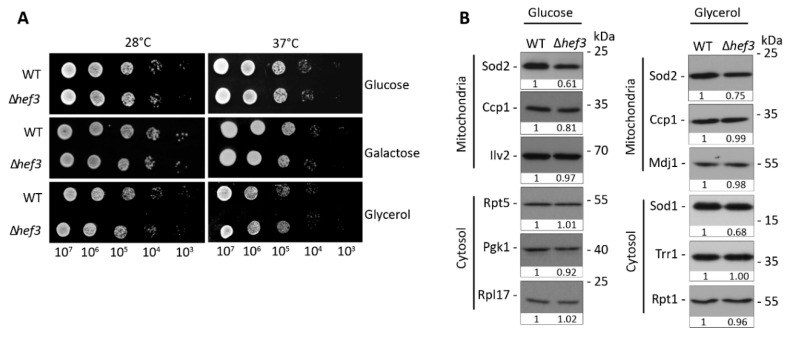
Cells that were deleted of *HEF3* gene were superficially wild-type under normal growth conditions. (**A**) Serial dilutions of wild-type yeast cells and *hef3*Δ cells were spotted on agar plates that contained different carbon sources as indicated. Yeast cells were grown for three days at different growth temperatures. (**B**) Wild-type yeast cells and *hef3*Δ cells were grown on fermentative medium (glucose) or respiratory medium (glycerol) at 28 °C. Total protein extracts were separated by SDS-PAGE and analyzed by Western blot using specific antibodies. The Western blot analysis was repeated in two independent experiments for each growth condition. Densitometry analysis of Western blot signals were performed and are shown below each blot as fold change compared to the protein levels in wild-type cells.

The two paralogs of yeast eEF3, Yef3p and Hef3p, share 84% sequence identity in a pairwise alignment. However, endogenous Hef3p cannot substitute for the essential functions of Yef3p. Consequently, cells that harbor a *YEF3* deletion are not viable. Only when *HEF3* was expressed under control of the endogenous promoter of *YEF3* could the lethal phenotype be rescued upon *YEF3* deletion [28,29]. This could indicate that regulation of the two paralogs under different growth conditions defines their functional activity. Thus, we investigated whether Hef3p is necessary for yeast growth under certain carbon source and temperature conditions. In contrast to *yef3*Δ cells, *hef3*Δyeast cells are viable. We performed a yeast growth test in BY4741wild-type cells and *hef3*Δ cells with chromosomal *HEF3* deletion. Yeast cells that were cultured on agar plates were used to prepare serial dilutions that were then spotted on solid plates and incubated at different growth temperatures (Figure 1A).

We did not observe any defect in the growth of *hef3*Δ cells compared with wild-type cells. This indicates that Hef3p is not required under basal growth conditions (i.e., neither fermentative nor respiratory conditions). eEF3 is involved in protein synthesis. Therefore, we also analyzed steady-state levels of selected proteins under fermentative and respiratory growth conditions (Figure 1B). The analyzed proteins localize to mitochondria or cytosol. The analyzed proteins were similar in their levels between *hef3*Δ and wild-type cells, which is in agreement with the growth assay. However, we noticed a decrease in superoxide dismutase 2 (Sod2p) protein levels in *hef3*Δ cells compared with wild-type cells, which was independent of the growth condition. Sod2p is a mitochondrial matrix protein and necessary for converting toxic superoxide byproducts of oxidative phosphorylation into less reactive H_2_O_2_.

### 3.2. Deletion of HEF3 Results in Growth Defect upon Oxidative Stress

**Figure 2:**
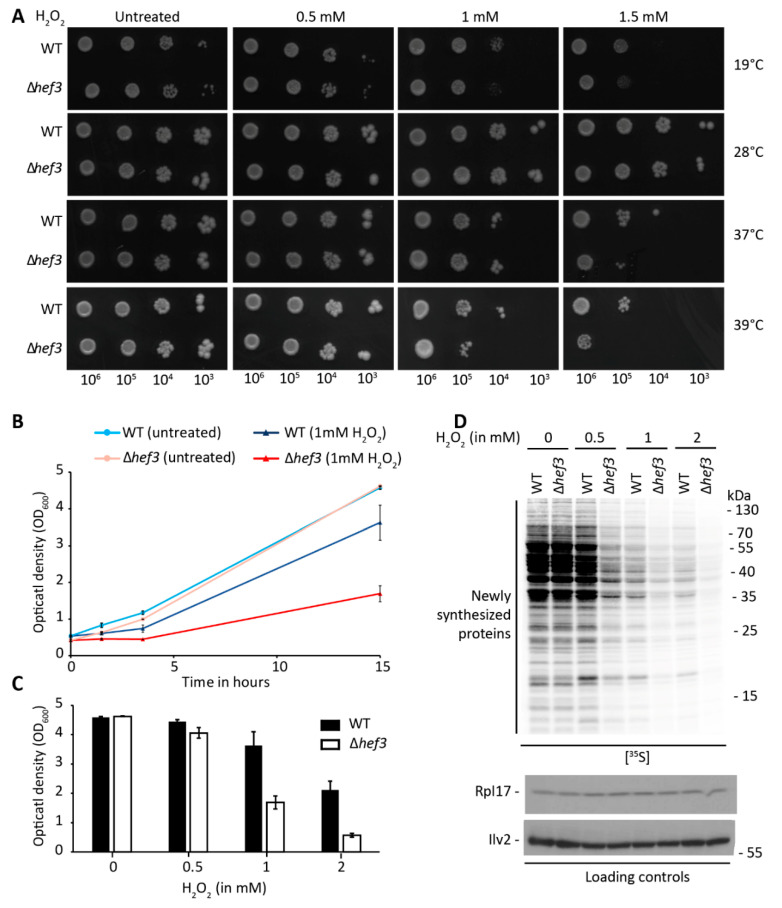
Deletion of the *HEF3* gene sensitizes yeast cells to oxidative stress. (**A**) Serial dilutions of wild-type yeast cells and *hef3*Δ cells were grown for five days on solid medium with a fermentative carbon source (sucrose) that contained the indicated concentrations of H_2_O_2_ or were untreated at different temperatures. The growth test was repeated in three independent experiments. (**B**,**C**) Cells were grown at 28 °C in a synthetic defined medium that contained glucose to the logarithmic growth phase. (**B**) H_2_O_2_ (1 mM) was added, and optical density was monitored over time. The mean ± SD of at least two independent experiments is presented. (**C**) The indicated concentrations of H_2_O_2_ were added, and cell growth was monitored. The graph represents the optical density after 15 h of growth. The data are expressed as the mean ± SD. The experiment was repeated at least two times. (**D**) Cells at the logarithmic growth phase were incubated for 30 min with the indicated concentrations of H_2_O_2_, and newly synthesized proteins were simultaneously labeled with [^35^S]-labeled methionine. Total cell extracts were separated by SDS-PAGE and analyzed by autoradiography or Western blot using specific antibodies. The analysis was repeated in three independent experiments.

Following our initial observation that cells that were deleted of *HEF3* had mild defects in expression of the ROS-detoxifying enzyme Sod2p, we analyzed the growth of *hef3*Δ cells under oxidative stress conditions by adding H_2_O_2_ exogenously to the cells. Serial dilutions of stationary-phase cultures of wild-type and *hef3*Δ cells were spotted on agar plates supplemented with 0.5, 1, or 1.5 mM H_2_O_2_ and incubated at different growth temperatures (Figure 2A). The growth of untreated cells did not differ between wild-type and *hef3*Δ cells, but a growth defect of *hef3*Δ cells compared with wild-type cells was observed upon treatment with H_2_O_2_. This growth defect was especially visible on plates that contained 1 or 1.5 mM H_2_O_2_ and when cells were grown at higher growth temperatures (Figure 2A). The resistance of cells to the lower concentration of H_2_O_2_ was likely attributable to their stationary growth phase, which makes yeast cells generally more resistant to stress [37]. Thus, we also analyzed the influence of oxidative stress on the growth of *hef3*Δ cells in liquid medium (Figure 2B). Yeast cells were grown to the logarithmic growth phase, and 1 mM H_2_O_2_ was added to the cultures. The optical density was then measured over time. The growth of wild-type cells was only mildly impaired upon the addition of H_2_O_2_, whereas *hef3*Δ cells grew significantly slower. We did not detect a difference in growth in cultures that were grown in parallel without the addition of H_2_O_2_ (Figure 2B). We tested the effect of further concentrations of H_2_O_2_ on logarithmically grown wild-type and *hef3*Δ cells and found that 0.5 mM H_2_O_2_ only mildly impaired growth, whereas 2 mM H_2_O_2_ inhibited the growth of *hef3*Δ cells entirely (Figure 2C).

Next, we investigated whether the growth impairments were attributable to lower levels of cytoplasmic translation. Wild-type cells and *hef3*Δ cells were labeled with radioactive methionine while grown with or without H_2_O_2_. Total cells extracts were analyzed by autoradiography (Figure 2D). The deletion of *HEF3* did not change the levels of global cytoplasmic translation under non-stressed growth conditions. Oxidative stress is known to result in the attenuation of translation [38,39], which was concentration-dependently observed in wild-type cells with the exogenous addition of H_2_O_2_. However, the deletion of *HEF3* gene accelerated this response, in which we observed a strong decrease in the signal for newly synthesized proteins already at a concentration of 0.5 mM H_2_O_2_, despite only mild growth impairment (Figure 2C). These findings indicate that Hef3p function is necessary to withstand oxidative stress, and this function cannot be compensated by Yef3p function alone.

### 3.3. HEF3 Expression Is Regulated by Oxidative Stress Conditions

**Figure 3:**
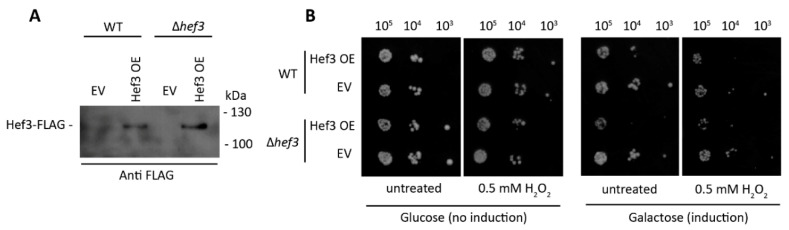
Ectopic expression of Hef3p negatively affects cell growth under both standard conditions and oxidative stress conditions. (**A**) FLAG-tagged Hef3 under the control of galactose inducible promoter (Hef3 OE) expression was induced for 4.5 h on a defined synthetic medium that contained 2% galactose in wild-type cells and *hef3*Δ cells. Total cell extracts were separated by SDS-PAGE and analyzed by Western blot using anti-FLAG antibody. (**B**) Wild-type cells or *hef3Δ* cells were transformed with an empty vector (EV) or FLAG-tagged Hef3. Serial dilutions of transformed cells were spotted on agar plates with glucose (no induction) or galactose (induction of Hef3 expression) supplemented or not with H_2_O_2_. Cells were grown for three days at 28 °C.

To investigate whether the ectopic expression of Hef3p is beneficial for yeast cells under oxidative stress conditions, we generated a construct that expressed Hef3p tagged at the C-terminus with FLAG-tag under control of the galactose inducible promoter. Wild-type and *hef3*Δ cells were transformed, and the expression of Hef3-FLAG was confirmed by Western blot (Figure 3A).

**Figure 4:**
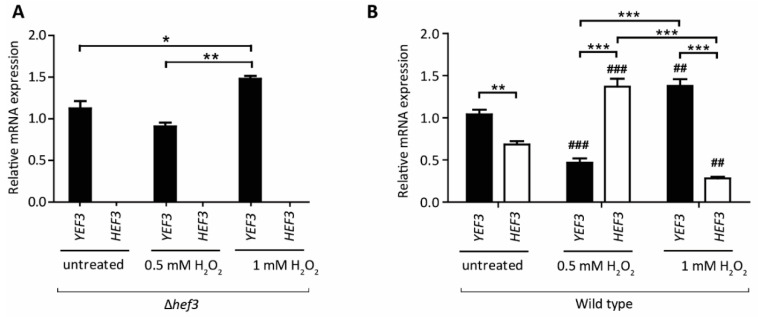
*HEF3* transcript levels are regulated under conditions of oxidative stress (**A**) Cells deleted of *HEF3* were grown in a synthetic defined medium that contained 2% glucose to the logarithmic growth phase at 28 °C. Transcript levels of *YEF3* and *HEF3* were evaluated by quantitative real-time PCR. The data are expressed as mean ± SEM. At least three biological replicates per condition were performed. * *p* < 0.05, ** *p* < 0.01. (**B**) Wild-type cells were grown on a fermentative medium at 28 °C to the logarithmic growth phase and treated with 0.5 or 1 mM H_2_O_2_ for 30 min or left untreated. Total RNA was isolated and analyzed for *YEF3* and *HEF3* mRNA. The data are expressed as mean ± SEM. At least three biological replicates per condition were performed. ** *p* < 0.01, *** *p* < 0.001. ^##^ *p* < 0.01, ^###^ *p* < 0.001, significance of the transcript level upon H_2_O_2_-treated cells compared to the same analyzed transcript of the corresponding untreated cells.

Next, we performed a growth assay of wild-type cells and *hef3*Δ cells, each expressing FLAG-tagged Hef3 (Hef3 OE) or an empty vector (EV) control (Figure 3B). Surprisingly, we found that Hef3p overexpression negatively influenced yeast growth already in non-stressed conditions. We observed the stronger sensitivity of cells that overexpressed Hef3p compared with the EV control under mild oxidative stress conditions. The growth defect upon Hef3p overexpression was independent of oxidative stress. Therefore, we concluded that the endogenous promoter of *HEF3* might be tightly controlled within the cell. To investigate whether *HEF3* expression at the transcript level is influenced by oxidative stress, we performed quantitative real-time PCR. The primers that were used to amplify the *HEF3* transcript were specific. No product of *HEF3* was detected in cells with the chromosomal deletion of *HEF3* (Figure 4A). First, we analyzed *YEF3* mRNA in *hef3*Δ cells in non-stressed conditions and after H_2_O_2_ treatment to rule out a potential compensatory effect for the loss of *HEF3*. *YEF3* mRNA levels did not significantly change in *hef3*Δ upon mild oxidative stress (0.5 mM H_2_O_2_) but increased under harsher stress conditions (Figure 4A). This result might indicate that the intensity of the stress influences the transcriptional response differentially.

Next, we analyzed *YEF3* and *HEF3* mRNA levels in wild-type cells grown on a fermentative medium. We detected mRNA levels of the both paralogs, although in non-stressed condition *HEF3* expression was lower than *YEF3* mRNA levels (Figure 4B). Upon treatment of cells with 0.5 mM H_2_O_2_ *HEF3* mRNA levels significantly increased by 91% whereas *YEF3* transcript levels decreased by 56% (Figure 4B). In contrast, when cells were treated with 1 mM H_2_O_2_ *YEF3* mRNA levels increased, similar to *hef3*Δ cells, but *HEF3* mRNA levels decreased by 56% (Figure 4B). This supports the assumption that *HEF3* gene expression is tightly controlled under both non-stressed growth conditions [28] and oxidative stress conditions. Our experiments suggest that *HEF3* is upregulated under mild oxidative stress conditions but more severe conditions favor the expression of *YEF3*.

### 3.4. Hef3p Is Necessary for the Expression of Oxidative Stress Response Proteins at the Translational Level

**Figure 5:**
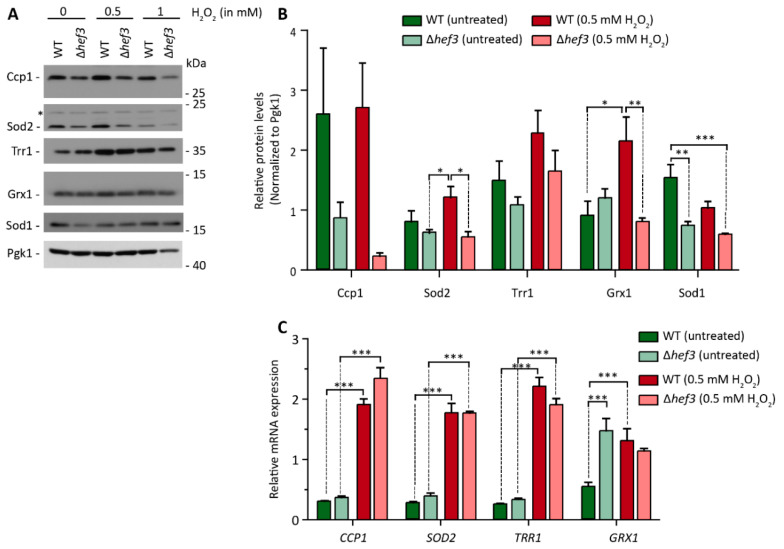
Levels of oxidative stress-response proteins depend on Hef3p. (**A**) Wild-type cells and *hef3*Δ cells were grown on fermentative medium to the logarithmic growth phase and treated with the indicated concentrations of H_2_O_2_ for 30 min or left untreated. Total cell extracts were separated by SDS-PAGE and analyzed by Western blot using specific antibodies. *, non-specific band. (**B**) Quantification of protein levels from four biological replicates. Data are presented as mean ± SEM. * *p* < 0.05, ** *p* < 0.01, *** *p* < 0.001. (**C**) Wild-type cells and *hef3*Δ cells were grown on fermentative medium to the logarithmic growth phase and treated with 0.5 mM H_2_O_2_ for 30 min or left untreated. Total RNA was isolated and analyzed for *SOD2*, *CCP1*, *TRR1*, and *GRX1* mRNA. The data are expressed as the mean ± SEM. *n* = 4. *** *p* < 0.001. Under more severe stress conditions (1 mM H_2_O_2_), protein levels decreased overall in *hef3*Δ cells, even for the control protein Pgk1, which is likely a consequence of the strong inhibition of global protein synthesis (see also Figure 2D).

We initially found that cells that lacked Hef3p had lower steady-state protein levels of Sod2 (see also Figure 1B). We next investigated whether Hef3p influences the expression of proteins that are necessary for ROS detoxification upon oxidative stress. We treated wild-type cells and *hef3*Δ cells with 0.5 or 1 mM H_2_O_2_ and analyzed protein levels of ROS-detoxifying proteins in mitochondria (i.e., Ccp1 and Sod2) and the cytoplasm (i.e., Trr1, Grx1, and Sod1; Figure 5A). The changes in the protein levels in the course of the experiment were quantified and normalized to the expression of Pgk1p, which is not involved ROS defense, in wild-type and *hef3*Δ cells (Figure 5B). Under mild oxidative stress conditions (0.5 mM H_2_O_2_), wild-type cells tended to exhibit an increase in the levels of ROS-detoxifying enzymes compared to non-stressed conditions. This was especially observed for Sod2p, Trr1p, and Grx1p. In contrast, in *hef3*Δ cells, the levels of proteins remained lower compared to wild-type cells upon mild oxidative stress (i.e., Ccp1p, Sod2p, and Grx1p).

Next, we investigated whether the differences in the protein levels of ROS-detoxifying enzymes in *hef3*Δ cells were a consequence of differences at the transcriptional level or translational level. We analyzed transcript levels of *CCP1*, *SOD2*, *TRR1*, and *GRX1* in wild-type and *hef3*Δ cells under non-stressed conditions and mild oxidative stress conditions (0.5 mM H_2_O_2_; Figure 5C). In wild-type cells, relative transcript levels in untreated cells were low and comparable but increased significantly upon oxidative stress. Similarly, in *hef3*Δ cells, *CCP1*, *SOD2*, and *TRR1* transcript levels increased upon oxidative stress, and we did not observe a significant difference between their increase in *hef3*Δ cells compared with wild-type cells. Interestingly, relative transcript levels of *GRX1* already increased under non-stressed conditions in *hef3*Δ cells compared with wild-type cells, indicating that *HEF3* deletion causes stress for which yeast cells attempt to compensate by adapting transcription. *GRX1* mRNA levels did not further increase upon oxidative stress. The increase in *GRX1* transcript levels in *hef3*Δ cells under basal conditions were not reflected by protein levels. In wild-type cells under stressed conditions Grx1p levels increased significantly, which correlated with the increased *GRX1* transcript levels (compare Figure 5B,C). The significant increases in *CCP1* and *SOD2* transcript levels in *hef3*Δ cells upon 0.5 mM H_2_O_2_ treatment did not reflect protein levels (compare Figure 5B,C). Trr1 protein levels showed a tendency to increase in *hef3*Δ cells upon oxidative stress but not to the same extent as in wild-type cells. Thus, Hef3p appears to be necessary for cellular defense against oxidative stress by contributing to the expression of ROS-detoxifying enzymes, and this expression is regulated at the translational levels rather than at the transcriptional level.

## 4. Discussion

The synthesis of new proteins is vital to all cellular functions but needs to be tightly regulated to adjust to intrinsic and extrinsic factors. The modulation of translation is crucial for maintaining cellular protein homeostasis. Imbalances in protein homeostasis can have detrimental effects on cellular function and the health of the organism [40,41]. The regulation of gene expression upon cellular stress via the adjustment of transcription has been widely investigated [42,43], but it does not always reflect the actual amount of proteins that is produced [44]. Thus, the regulation of protein expression at the post-transcriptional level has wide implications for the cellular response to stress [39,45,46]. An increasing number of reports has identified various ribosome-associated proteins that can influence translational output in a given cellular environment or under certain stress conditions [47]. The *HEF3* gene is a paralog of the essential gene *YEF3*, which encodes eEF3 in yeast, and in contrast to *HEF3* is indispensable for translation. Two other elongation factors, eEF1 and eEF2, are also encoded by paralogous genes in yeast (*TEF1*/*TEF2* and *EFT1*/*EFT2*, respectively). However, the paralogous genes that encode eEF1 and eEF2 can substitute for each other; thus, only the deletion of both paralogs is lethal for the cell. This is in contrast to eEF3 and suggests that *HEF3* has a function that is independent of *YEF3*. In the present study, we identified the translation elongation factor Hef3 as a protein that is necessary to help the cell cope with oxidative stress. Oxidative stress is a well-understood regulator of gene transcription [48]. Consistent with previous findings, real-time PCR revealed a robust increase in transcript levels of genes that encode ROS-defense proteins upon the treatment of cells with H_2_O_2_ (Figure 5C). This increase in mRNA levels was independent of *HEF3*. However, higher mRNA levels in *hef3*Δ cells did not result in a consistent increase in protein levels (Figure 5A). Thus, our data implicate Hef3p in regulation of the production of ROS-defense proteins at the translational level. Our findings provide further evidence of a cellular function of Hef3p.

Translation adaptation upon oxidative stress occurs at both the initiation phase and the elongation phase [39]. The ways in which Hef3p mediates the expression of ROS-defense proteins remains to be determined. Hef3p was shown to have similar affinity for the ribosome as its paralog Yef3p [28]. Yef3p stimulates the eEF1A-mediated binding of cognate aa-tRNA to the A-site [18,49]. In yeast, a mechanism was proposed whereby exposure to H_2_O_2_ results in hypermodified tRNA^Leu(CAA)^, which in turn favors the production of alternative paralogous ribosomal proteins [50]. The incorporation of these alternative ribosomal proteins in the ribosome resulted in the selective expression of proteins from TTG-enriched genes [50,51]. One possible scenario could be that Hef3p is involved in the mediation of binding of modified tRNAs to the A-site, but such a possibility requires further investigation.

The *HEF3* gene is not required for yeast viability and fitness under basal growth conditions, which raises a question about the regulation of *HEF3* gene expression. In contrast to other studies, we detected the expression of *HEF3* under standard yeast growth conditions using quantitative real-time PCR, which might have provided a more sensitive readout than Northern blot analysis. However, this does not imply that a functional protein is produced. The overexpression of Hef3p had a dominant-negative effect on yeast growth (Figure 3B), suggesting that *HEF3* expression from the endogenous promoter is tightly regulated. Previous Northern blot analysis showed that the expression of *HEF3* could only be detected under zinc-deficient growth conditions, but the role of Hef3p in the cell remained elusive [30]. Zinc is an essential cofactor of many proteins, and zinc deficiency leads to a slowdown of transcription and translation [52]. Zinc-finger-like motifs were also found in ribosomal proteins. Interestingly, ribosomal proteins with a zinc-binding motif were shown in a proteome-wide analysis to be redox-sensitive [38]. Biological consequences of ribosomal protein oxidation remain to be described but will be interesting in future investigations of potential relationships between the oxidation of ribosomal proteins, their zinc-binding ability, and Hef3p function. Interestingly, we found that *HEF3* mRNA levels were increased upon mild oxidative stress while *YEF3* levels were decreased (Figure 3D). In a study that analyzed genome-wide binding locations of transcription-related proteins, it was found that Skn7p can regulate *HEF3* transcription [53]. Skn7p is a major transcription factor in yeast regulating transcript levels of oxidative-stress defense genes. This proposed regulation of *HEF3* expression by Skn7p has not been validated in an independent approach. However, it does support our hypothesis that Hef3p function is stimulated by oxidative stress.

80S ribosomes of non-fungal eukaryotes were proposed to have an intrinsic ATPase that fulfils the function of eEF3. Therefore, eEF3 became a ribosomal protein or at least a ribosomal component during the course of evolution [54]. The Rli1/ABCE1 protein is conserved in Archaea and mammals, exhibits ATPase activity, and is involved in ribosome recycling, ribosome biogenesis, and translation initiation [23,55,56]. The gene that encodes Rli1/ABCE1 is also essential for life in yeast. Thus, yeast were proposed to be supplemented with a presumably primordial eEF3 function [57]. However, Rli1/ABCE1 contains an iron-sulfur cluster domain [58,59] and is inactivated upon mild oxidative stress [60]. Remaining to be elucidated is whether a ribosomal factor that is similar to Hef3p is involved in the modulation of translation elongation upon oxidative stress in non-fungal species.

## 5. Conclusions

Fungi require the eEF3 for the synthesis of proteins. The eEF3 is encoded by two paralogous genes *YEF3* and *HEF3*. While Yef3p is essential in yeast during protein synthesis the function of its paralog Hef3p remained elusive. Here, for the first time, we show that Hef3p is required for yeast growth under oxidative stress conditions. Protein synthesis is unaffected under non-stressed conditions in the absence of *HEF3* but the loss of Hef3p function under oxidative stress conditions led to accelerated translation attenuation and consequently in yeast growth defect. We showed that *HEF3* transcript levels were increased upon mild oxidative stress. Subsequently, Hef3p is necessary for the expression of ROS detoxifying proteins at the translational level rather than transcriptional. This newly described function of *HEF3* is at least in part independent of its paralog *YEF3* because *YEF3* transcript levels were not altered in *hef3*Δ cells under mild oxidative stress conditions. Therefore, we propose that Hef3p acts as a ribosome-associated protein that can modulate translational output upon cellular conditions that increase oxidative stress.

## Figures and Tables

**Table 1:** Sense and antisense primers that were used in this study.

Gene	Forward (5′–3′)	Reverse (5′–3′)	Reference
*HEF3*	CGCTAAAGAACAGATTGCCT	TTCAGTTGCCTTTGTGATGG	Present study
*YEF3*	GCTATCTCTGCTATGGTCGA	CAGCCTTGACTTCCTTCTTG	
*CCP1*	ACAACGAACAGTGGGACTCT	ACTTGTCCTGGTCATTAGCGT	
*TRR1*	CCGTCCCCATTTTCAGAAACA	GCACGCAAATGGTCTTTTCTG	
*SOD2*	CTCTAGTTGCCATTGACGCC	TGCCAGCATCGAATCTTCTG	
*GRX1*	CGTCGCATCCAAAACGTACT	GCGCCTTCCTTCATGTCATT	
*ACT1*	CATGTTCCCAGGTATTGCCG	GTCAAAGAAGCCAAGATAGA	[36]
*ALG9*	TCCATGATACAGGAGCAAGC	CTACCATCAGAACCGCATTC	
*TDH1*	GGTATGGCTTTCAGAGTCCCA	AGACAACGGCATCTTCGGTG	
